# Supplementary material for: The benefits and harms of adjuvant chemotherapy for non-small cell lung cancer in patients with major comorbidities: A simulation study
Source: PLoS One. 2022 Nov 15;17(11):e0263911. doi: 10.1371/journal.pone.0263911 (PMC9665372; doi:10.1371/journal.pone.0263911)
Supplement: S1 Table — (DOCX) [file pone.0263911.s001.docx]

|  |  | |  | |  | |  |  | |  | |  | |  |  | |  |
| --- | --- | --- | --- | --- | --- | --- | --- | --- | --- | --- | --- | --- | --- | --- | --- | --- | --- |
| **S1 Table.** Estimated quality-adjusted life expectancy according to patient group, treatment and comorbidity | | | | | | | | | | | | | | | | | |
| **Patient Category** | **Treatment** | **QALY Gains by Comorbidities** | | | | | | | | | | | | | | | |
|  |  | **None** | | **CAD** | | **CHF** | | | **COPD** | | **CAD/CHF** | | **CHF/COPD** | | | **CAD/COPD** | |
| 80-84 years, Male, Stage IB | Adjuvant Chemotherapy | 5.8 | | 5.2 | | 4.8 | | | 4.7 | | 4.1 | | 3.9 | | | 4.2 | |
|  | Observation | 5.6 | | 5.1 | | 4.7 | | | 4.6 | | 4.2 | | 3.8 | | | 4.1 | |
| 80-84 years, Male, Stage IIA | Adjuvant Chemotherapy | 5.6 | | 5.1 | | 4.6 | | | 4.6 | | 4.0 | | 3.7 | | | 4.1 | |
|  | Observation | 5.4 | | 4.9 | | 4.6 | | | 4.4 | | 4.1 | | 3.7 | | | 4.0 | |
| 80-84 years, Male, Stage IIB | Adjuvant Chemotherapy | 4.7 | | 4.3 | | 3.9 | | | 3.7 | | 3.5 | | 3.1 | | | 3.3 | |
|  | Observation | 4.3 | | 4.0 | | 3.8 | | | 3.4 | | 3.4 | | 2.9 | | | 3.1 | |
| 80-84 years, Male, Stage IIIA | Adjuvant Chemotherapy | 3.6 | | 3.3 | | 3.1 | | | 2.7 | | 2.7 | | 2.3 | | | 2.5 | |
|  | Observation | 3.2 | | 3.0 | | 2.9 | | | 2.4 | | 2.6 | | 2.1 | | | 2.3 | |
| 75-79 years, Male, Stage IB | Adjuvant Chemotherapy | 7.0 | | 6.2 | | 5.7 | | | 5.7 | | 5.0 | | 4.6 | | | 5.1 | |
|  | Observation | 6.7 | | 6.0 | | 5.6 | | | 5.4 | | 4.9 | | 4.5 | | | 4.9 | |
|  |  | **None** | | **CAD** | | **CHF** | | | **COPD** | | **CAD/CHF** | | **CHF/COPD** | | | **CAD/COPD** | |
| 75-79 years, Male, Stage IIA | Adjuvant Chemotherapy | 6.8 | | 6.1 | | 5.6 | | | 5.5 | | 4.8 | | 4.4 | | | 4.9 | |
|  | Observation | 6.4 | | 5.8 | | 5.4 | | | 5.1 | | 4.8 | | 4.3 | | | 4.7 | |
| 75-79 years, Male, Stage IIB | Adjuvant Chemotherapy | 5.6 | | 5.1 | | 4.7 | | | 4.4 | | 4.1 | | 3.6 | | | 3.9 | |
|  | Observation | 5.1 | | 4.7 | | 4.4 | | | 4.0 | | 4.0 | | 3.4 | | | 3.6 | |
| 75-79 years, Male, Stage IIIA | Adjuvant Chemotherapy | 4.2 | | 3.9 | | 3.6 | | | 3.1 | | 3.2 | | 2.7 | | | 2.9 | |
|  | Observation | 3.8 | | 3.5 | | 3.3 | | | 2.7 | | 3.0 | | 2.4 | | | 2.6 | |
| 70-74 years, Male, Stage IB | Adjuvant Chemotherapy | 8.2 | | 7.3 | | 6.6 | | | 6.7 | | 5.7 | | 5.3 | | | 5.9 | |
|  | Observation | 7.8 | | 7.0 | | 6.5 | | | 6.3 | | 5.7 | | 5.2 | | | 5.6 | |
| 70-74 years, Male, Stage IIA | Adjuvant Chemotherapy | 7.9 | | 7.1 | | 6.4 | | | 6.4 | | 5.6 | | 5.2 | | | 5.7 | |
|  | Observation | 7.5 | | 6.8 | | 6.3 | | | 6.0 | | 5.5 | | 5.0 | | | 5.4 | |
| 70-74 years, Male, Stage IIB | Adjuvant Chemotherapy | 6.6 | | 5.9 | | 5.4 | | | 5.1 | | 4.8 | | 4.2 | | | 4.6 | |
|  | Observation | 6.1 | | 5.5 | | 5.1 | | | 4.7 | | 4.6 | | 3.9 | | | 4.2 | |
| 70-74 years, Male, Stage IIIA | Adjuvant Chemotherapy | 5.0 | | 4.5 | | 4.2 | | | 3.7 | | 3.7 | | 3.1 | | | 3.0 | |
|  | Observation | 4.4 | | 4.1 | | 3.8 | | | 3.2 | | 3.5 | | 2.8 | | | 3.4 | |
|  |  | **None** | | **CAD** | | **CHF** | | | **COPD** | | **CAD/CHF** | | **CHF/COPD** | | | **CAD/COPD** | |
| 66-69 years, Male, Stage IB | Adjuvant Chemotherapy | 9.8 | | 8.7 | | 7.9 | | | 8.1 | | 6.8 | | 6.4 | | | 7.1 | |
|  | Observation | 9.4 | | 8.4 | | 7.7 | | | 7.6 | | 6.8 | | 6.2 | | | 6.8 | |
| 66-69 years, Male, Stage IIA | Adjuvant Chemotherapy | 9.6 | | 8.5 | | 7.7 | | | 7.8 | | 6.6 | | 6.2 | | | 6.9 | |
|  | Observation | 9.1 | | 8.2 | | 7.5 | | | 7.3 | | 6.7 | | 6.0 | | | 6.6 | |
| 66-69 years, Male, Stage IIB | Adjuvant Chemotherapy | 8.1 | | 7.3 | | 6.6 | | | 6.4 | | 5.8 | | 5.2 | | | 5.7 | |
|  | Observation | 7.5 | | 6.8 | | 6.3 | | | 5.8 | | 5.5 | | 4.8 | | | 5.2 | |
| 66-69 years, Male, Stage IIIA | Adjuvant Chemotherapy | 6.3 | | 5.7 | | 5.2 | | | 4.7 | | 4.6 | | 3.9 | | | 4.2 | |
|  | Observation | 5.6 | | 5.1 | | 4.8 | | | 4.1 | | 4.3 | | 3.5 | | | 3.7 | |
| 80-84 years, Female, Stage IB | Adjuvant Chemotherapy | 6.7 | | 6.1 | | 5.7 | | | 5.6 | | 5.0 | | 4.7 | | | 5.1 | |
|  | Observation | 6.5 | | 6.0 | | 5.6 | | | 5.4 | | 5.0 | | 4.6 | | | 5.0 | |
| 80-84 years, Female, Stage IIA | Adjuvant Chemotherapy | 6.6 | | 6.0 | | 5.5 | | | 5.5 | | 4.9 | | 4.6 | | | 5.0 | |
|  | Observation | 6.3 | | 5.8 | | 5.5 | | | 5.2 | | 4.9 | | 4.5 | | | 4.8 | |
| 80-84 years, Female, Stage IIB | Adjuvant Chemotherapy | 5.6 | | 5.1 | | 4.8 | | | 4.5 | | 4.3 | | 3.8 | | | 4.1 | |
|  | Observation | 5.3 | | 4.9 | | 4.6 | | | 4.2 | | 4.2 | | 3.6 | | | 3.9 | |
| 80-84 years, Female, Stage IIIA | Adjuvant Chemotherapy | 4.4 | | 4.1 | | 3.8 | | | 3.4 | | 3.4 | | 2.9 | | | 3.1 | |
|  | Observation | 4.0 | | 3.7 | | 3.5 | | | 3.0 | | 3.2 | | 2.7 | | | 2.8 | |
|  |  | **None** | | **CAD** | | **CHF** | | | **COPD** | | **CAD/CHF** | | **CHF/COPD** | | | **CAD/COPD** | |
| 75-79 years, Female, Stage IB | Adjuvant Chemotherapy | 8.1 | | 7.4 | | 6.8 | | | 6.8 | | 6.0 | | 5.6 | | | 6.1 | |
|  | Observation | 7.8 | | 7.2 | | 6.7 | | | 6.5 | | 6.0 | | 5.5 | | | 5.9 | |
| 75-79 years, Female, Stage IIA | Adjuvant Chemotherapy | 7.9 | | 7.2 | | 6.7 | | | 6.6 | | 5.9 | | 5.5 | | | 5.9 | |
|  | Observation | 7.6 | | 7.0 | | 6.5 | | | 6.2 | | 5.8 | | 5.3 | | | 5.7 | |
| 75-79 years, Female, Stage IIB | Adjuvant Chemotherapy | 6.3 | | 5.7 | | 5.3 | | | 5.0 | | 4.7 | | 4.2 | | | 4.6 | |
|  | Observation | 5.9 | | 5.4 | | 5.0 | | | 4.6 | | 4.5 | | 4.0 | | | 4.2 | |
| 75-79 years, Female, Stage IIIA | Adjuvant Chemotherapy | 5.2 | | 4.4 | | 4.5 | | | 3.8 | | 4.1 | | 3.4 | | | 3.6 | |
|  | Observation | 4.7 | | 4.8 | | 4.1 | | | 3.5 | | 3.8 | | 3.1 | | | 3.2 | |
| 70-74 years, Female, Stage IB | Adjuvant Chemotherapy | 9.6 | | 8.7 | | 8.0 | | | 8.0 | | 7.0 | | 6.6 | | | 7.2 | |
|  | Observation | 9.2 | | 8.4 | | 7.8 | | | 7.6 | | 6.9 | | 6.3 | | | 6.9 | |
| 70-74 years, Female, Stage IIA | Adjuvant Chemotherapy | 9.3 | | 8.4 | | 7.8 | | | 7.7 | | 6.8 | | 6.4 | | | 6.9 | |
|  | Observation | 8.9 | | 8.1 | | 7.6 | | | 7.3 | | 6.7 | | 6.1 | | | 6.6 | |
| 70-74 years, Female, Stage IIB | Adjuvant Chemotherapy | 8.0 | | 7.2 | | 6.7 | | | 6.4 | | 5.9 | | 5.3 | | | 5.7 | |
|  | Observation | 7.4 | | 6.8 | | 6.3 | | | 5.8 | | 5.7 | | 5.0 | | | 5.3 | |
| 70-74 years, Female, Stage IIIA | Adjuvant Chemotherapy | 6.2 | | 5.7 | | 5.3 | | | 4.7 | | 4.7 | | 4.0 | | | 4.3 | |
|  | Observation | 5.5 | | 5.1 | | 4.8 | | | 4.1 | | 4.4 | | 3.6 | | | 3.8 | |
| 66-69 years, Female, Stage IB | Adjuvant Chemotherapy | 11.5 | | 10.3 | | 9.5 | | | 9.7 | | 8.3 | | 7.9 | | | 8.6 | |
|  | Observation | 11.0 | | 10.0 | | 9.3 | | | 9.2 | | 8.2 | | 7.6 | | | 8.3 | |
| 66-69 years, Female, Stage IIA | Adjuvant Chemotherapy | 11.2 | | 10.1 | | 9.3 | | | 8.9 | | 8.2 | | 7.7 | | | 8.4 | |
|  | Observation | 10.7 | | 9.8 | | 9.1 | | | 8.2 | | 8.0 | | 7.4 | | | 8.0 | |
| 66-69 years, Female, Stage IIB | Adjuvant Chemotherapy | 9.7 | | 8.8 | | 8.1 | | | 7.8 | | 7.2 | | 6.5 | | | 7.1 | |
|  | Observation | 9.1 | | 8.3 | | 7.7 | | | 7.2 | | 6.9 | | 6.1 | | | 6.5 | |
| 66-69 years, Female, Stage IIIA | Adjuvant Chemotherapy | 7.7 | | 7.1 | | 6.6 | | | 5.9 | | 5.8 | | 5.0 | | | 5.4 | |
|  | Observation | 6.9 | | 6.4 | | 6.0 | | | 5.2 | | 5.4 | | 4.5 | | | 4.8 | |
