# Supplementary material for: The benefits and harms of adjuvant chemotherapy for non-small cell lung cancer in patients with major comorbidities: A simulation study
Source: PLoS One. 2022 Nov 15;17(11):e0263911. doi: 10.1371/journal.pone.0263911 (PMC9665372; doi:10.1371/journal.pone.0263911)
Supplement: S2 Table — (DOCX) [file pone.0263911.s002.docx]

|  |  | |  | |  |  |  |  |  |  |  | |
| --- | --- | --- | --- | --- | --- | --- | --- | --- | --- | --- | --- | --- |
| **S2 Table.** Estimated 5-year survival rates according to patient group, treatment and comorbidity | | | | | | | | | | | |  |
| **Patient Category** | **Treatment** | **5-Year Survival Rates (%) by Comorbidities** | | | | | | | | | |  |
|  |  | **None** | | **CAD** | | **CHF** | **COPD** | **CAD/CHF** | **CHF/COPD** | **CAD/COPD** | |  |
| 80-84 years, Male, Stage IB | Adjuvant Chemotherapy | 52 | | 49 | | 46 | 44 | 41 | 38 | 41 | |  |
|  | Observation | 50 | | 47 | | 45 | 42 | 41 | 37 | 39 | |  |
| 80-84 years, Male, Stage IIA | Adjuvant Chemotherapy | 51 | | 47 | | 44 | 43 | 40 | 36 | 39 | |  |
|  | Observation | 49 | | 46 | | 43 | 40 | 39 | 35 | 37 | |  |
| 80-84 years, Male, Stage IIB | Adjuvant Chemotherapy | 42 | | 39 | | 37 | 33 | 33 | 28 | 31 | |  |
|  | Observation | 39 | | 36 | | 35 | 30 | 32 | 26 | 28 | |  |
| 80-84 years, Male, Stage IIIA | Adjuvant Chemotherapy | 31 | | 29 | | 27 | 22 | 24 | 19 | 20 | |  |
|  | Observation | 27 | | 25 | | 24 | 18 | 22 | 16 | 17 | |  |
| 75-79 years, Male, Stage IB | Adjuvant Chemotherapy | 56 | | 53 | | 50 | 48 | 46 | 42 | 45 | |  |
|  | Observation | 54 | | 51 | | 49 | 46 | 46 | 41 | 43 | |  |
| 75-79 years, Male, Stage IIA | Adjuvant Chemotherapy | 55 | | 52 | | 49 | 47 | 45 | 41 | 44 | |  |
|  | Observation | 52 | | 50 | | 48 | 44 | 44 | 39 | 41 | |  |
| 75-79 years, Male, Stage IIB | Adjuvant Chemotherapy | 46 | | 43 | | 41 | 37 | 38 | 32 | 34 | |  |
|  | Observation | 42 | | 40 | | 38 | 33 | 36 | 29 | 31 | |  |
| 75-79 years, Male, Stage IIIA | Adjuvant Chemotherapy | 34 | | 32 | | 31 | 25 | 28 | 22 | 23 | |  |
|  | Observation | 30 | | 28 | | 27 | 21 | 25 | 18 | 19 | |  |
| 70-74 years, Male, Stage IB | Adjuvant Chemotherapy | 60 | | 57 | | 54 | 52 | 50 | 47 | 49 | |  |
|  | Observation | 58 | | 55 | | 53 | 49 | 48 | 45 | 47 | |  |
| 70-74 years, Male, Stage IIA | Adjuvant Chemotherapy | 58 | | 55 | | 53 | 50 | 49 | 45 | 47 | |  |
|  | Observation | 56 | | 53 | | 51 | 47 | 48 | 43 | 45 | |  |
| 70-74 years, Male, Stage IIB | Adjuvant Chemotherapy | 50 | | 47 | | 45 | 41 | 42 | 36 | 38 | |  |
|  | Observation | 46 | | 44 | | 42 | 37 | 40 | 33 | 35 | |  |
| 70-74 years, Male, Stage IIIA | Adjuvant Chemotherapy | 38 | | 36 | | 35 | 29 | 32 | 26 | 27 | |  |
|  | Observation | 33 | | 32 | | 31 | 24 | 29 | 22 | 23 | |  |
| 66-69 years, Male, Stage IB | Adjuvant Chemotherapy | 65 | | 62 | | 60 | 58 | 56 | 52 | 53 | |  |
|  | Observation | 63 | | 60 | | 58 | 55 | 55 | 51 | 51 | |  |
| 66-69 years, Male, Stage IIA | Adjuvant Chemotherapy | 63 | | 61 | | 58 | 56 | 55 | 51 | 52 | |  |
|  | Observation | 61 | | 59 | | 57 | 53 | 54 | 49 | 49 | |  |
| 66-69 years, Male, Stage IIB | Adjuvant Chemotherapy | 55 | | 53 | | 51 | 47 | 48 | 43 | 45 | |  |
|  | Observation | 52 | | 50 | | 48 | 43 | 46 | 40 | 41 | |  |
| 66-69 years, Male, Stage IIIA | Adjuvant Chemotherapy | 44 | | 42 | | 41 | 35 | 38 | 32 | 33 | |  |
|  | Observation | 40 | | 38 | | 37 | 30 | 35 | 28 | 29 | |  |
| 80-84 years, Female, Stage IB | Adjuvant Chemotherapy | 59 | | 56 | | 53 | 52 | 49 | 46 | 49 | |  |
|  | Observation | 57 | | 55 | | 53 | 49 | 49 | 44 | 46 | |  |
| 80-84 years, Female, Stage IIA | Adjuvant Chemotherapy | 58 | | 55 | | 52 | 50 | 48 | 44 | 47 | |  |
|  | Observation | 56 | | 53 | | 51 | 47 | 48 | 43 | 45 | |  |
| 80-84 years, Female, Stage IIB | Adjuvant Chemotherapy | 50 | | 47 | | 45 | 41 | 41 | 36 | 38 | |  |
|  | Observation | 46 | | 44 | | 42 | 37 | 40 | 33 | 35 | |  |
| 80-84 years, Female, Stage IIIA | Adjuvant Chemotherapy | 38 | | 36 | | 34 | 29 | 32 | 26 | 27 | |  |
|  | Observation | 33 | | 32 | | 31 | 25 | 29 | 22 | 23 | |  |
| 75-79 years, Female, Stage IB | Adjuvant Chemotherapy | 63 | | 60 | | 58 | 55 | 54 | 50 | 53 | |  |
|  | Observation | 61 | | 58 | | 56 | 53 | 53 | 48 | 50 | |  |
| 75-79 years, Female, Stage IIA | Adjuvant Chemotherapy | 61 | | 59 | | 56 | 54 | 53 | 49 | 51 | |  |
|  | Observation | 59 | | 57 | | 55 | 51 | 52 | 47 | 49 | |  |
| 75-79 years, Female, Stage IIB | Adjuvant Chemotherapy | 53 | | 51 | | 49 | 44 | 46 | 40 | 42 | |  |
|  | Observation | 49 | | 48 | | 46 | 40 | 43 | 37 | 39 | |  |
| 75-79 years, Female, Stage IIIA | Adjuvant Chemotherapy | 42 | | 40 | | 38 | 32 | 36 | 29 | 31 | |  |
|  | Observation | 37 | | 35 | | 34 | 27 | 32 | 25 | 26 | |  |
| 70-74 years, Female, Stage IB | Adjuvant Chemotherapy | 66 | | 63 | | 61 | 59 | 58 | 54 | 56 | |  |
|  | Observation | 64 | | 62 | | 60 | 56 | 57 | 52 | 54 | |  |
| 70-74 years, Female, Stage IIA | Adjuvant Chemotherapy | 65 | | 62 | | 60 | 57 | 57 | 53 | 55 | |  |
|  | Observation | 62 | | 60 | | 58 | 56 | 56 | 51 | 52 | |  |
| 70-74 years, Female, Stage IIB | Adjuvant Chemotherapy | 57 | | 54 | | 52 | 48 | 50 | 44 | 46 | |  |
|  | Observation | 53 | | 51 | | 50 | 44 | 47 | 41 | 42 | |  |
| 70-74 years, Female, Stage IIIA | Adjuvant Chemotherapy | 45 | | 44 | | 42 | 36 | 40 | 33 | 34 | |  |
|  | Observation | 41 | | 39 | | 38 | 31 | 36 | 29 | 30 | |  |
| 66-69 years, Female, Stage IB | Adjuvant Chemotherapy | 70 | | 68 | | 66 | 64 | 63 | 59 | 61 | |  |
|  | Observation | 68 | | 66 | | 65 | 62 | 62 | 58 | 60 | |  |
| 66-69 years, Female, Stage IIA | Adjuvant Chemotherapy | 69 | | 67 | | 65 | 62 | 62 | 58 | 60 | |  |
|  | Observation | 67 | | 65 | | 63 | 60 | 61 | 56 | 58 | |  |
| 66-69 years, Female, Stage IIB | Adjuvant Chemotherapy | 62 | | 60 | | 58 | 54 | 55 | 50 | 52 | |  |
|  | Observation | 59 | | 57 | | 55 | 50 | 53 | 47 | 49 | |  |
| 66-69 years, Female, Stage IIIA | Adjuvant Chemotherapy | 51 | | 50 | | 48 | 42 | 46 | 39 | 41 | |  |
|  | Observation | 47 | | 46 | | 44 | 37 | 42 | 35 | 36 | |  |
