# Supplementary material for: The benefits and harms of adjuvant chemotherapy for non-small cell lung cancer in patients with major comorbidities: A simulation study
Source: PLoS One. 2022 Nov 15;17(11):e0263911. doi: 10.1371/journal.pone.0263911 (PMC9665372; doi:10.1371/journal.pone.0263911)
Supplement: S3 Table — (DOCX) [file pone.0263911.s003.docx]

**S3 Table.** Percent of scenarios changing conclusions in sensitivity analyses: Varying lung cancer survival benefit over 95% confidence interval of hazard ratio

| **Stage IB** | **None** | **CAD** | **CHF** | **COPD** | **CAD/CHF** | **CHF/COPD** | **CAD/COPD** |
| --- | --- | --- | --- | --- | --- | --- | --- |
| Male 80-84 years | 44% | 22% | 0% | 44% | 0% | 0% | 22% |
| Male 75-79 years | 33% | 44% | 22% | 33% | 0% | 22% | 44% |
| Male 70-74 years | 22% | 56% | 33% | 22% | 11% | 33% | 56% |
| Male 66-69 years | 22% | 67% | 44% | 22% | 22% | 44% | 33% |
| Female 80-84 years | 44% | 22% | 0% | 56% | 0% | 11% | 33% |
| Female 75-79 years | 33% | 56% | 33% | 33% | 11% | 33% | 56% |
| Female 70-74 years | 22% | 67% | 44% | 22% | 22% | 44% | 33% |
| Female 66-69 years | 22% | 33% | 56% | 11% | 33% | 56% | 33% |
|  |  |  |  |  |  |  |  |
| **Stage IIA** | **None** | **CAD** | **CHF** | **COPD** | **CAD/CHF** | **CHF/COPD** | **CAD/COPD** |
| Male 80-84 years | 44% | 22% | 0% | 44% | 0% | 0% | 22% |
| Male 75-79 years | 33% | 56% | 33% | 33% | 0% | 33% | 56% |
| Male 70-74 years | 22% | 56% | 44% | 22% | 11% | 44% | 56% |
| Male 66-69 years | 22% | 33% | 56% | 11% | 22% | 56% | 33% |
| Female 80-84 years | 56% | 33% | 11% | 56% | 0% | 11% | 33% |
| Female 75-79 years | 33% | 56% | 44% | 22% | 11% | 44% | 56% |
| Female 70-74 years | 22% | 33% | 56% | 22% | 22% | 56% | 33% |
| Female 66-69 years | 11% | 33% | 56% | 11% | 33% | 56% | 22% |
|  |  |  |  |  |  |  |  |
| **Stage IIB** | **None** | **CAD** | **CHF** | **COPD** | **CAD/CHF** | **CHF/COPD** | **CAD/COPD** |
| Male 80-84 years | 33% | 44% | 33% | 67% | 0% | 22% | 44% |
| Male 75-79 years | 22% | 33% | 56% | 22% | 33% | 44% | 33% |
| Male 70-74 years | 11% | 22% | 33% | 11% | 44% | 56% | 22% |
| Male 66-69 years | 0% | 11% | 33% | 11% | 56% | 33% | 22% |
| Female 80-84 years | 33% | 56% | 44% | 33% | 22% | 33% | 56% |
| Female 75-79 years | 11% | 22% | 33% | 11% | 44% | 56% | 22% |
| Female 70-74 years | 11% | 11% | 33% | 11% | 56% | 33% | 22% |
| Female 66-69 years | 0% | 11% | 22% | 0% | 67% | 22% | 11% |
|  |  |  |  |  |  |  |  |
| **Stage IIIA** | **None** | **CAD** | **CHF** | **COPD** | **CAD/CHF** | **CHF/COPD** | **CAD/COPD** |
| Male 80-84 years | 22% | 56% | 44% | 33% | 22% | 33% | 44% |
| Male 75-79 years | 11% | 22% | 33% | 22% | 44% | 56% | 33% |
| Male 70-74 years | 0% | 11% | 22% | 11% | 56% | 33% | 22% |
| Male 66-69 years | 0% | 11% | 11% | 0% | 33% | 22% | 11% |
| Female 80-84 years | 22% | 33% | 56% | 22% | 44% | 44% | 33% |
| Female 75-79 years | 0% | 11% | 22% | 11% | 56% | 33% | 22% |
| Female 70-74 years | 0% | 11% | 11% | 0% | 33% | 22% | 11% |
| Female 66-69 years | 0% | 6% | 11% | 0% | 22% | 11% | 0% |
|  |  |  | |  |  |  |  |
| Base-case conclusion | (no color) | Observation |  | Adjuvant chemotherapy | | | |

*One-way sensitivity analyses show results of simulations varying parameter for impact of adjuvant chemotherapy on lung cancer-specific survival. Result is the proportion of simulations where the base-case prediction is maintained when varying the hazard ratio for adjuvant chemotherapy across its 95% confidence range.
