# Supplementary material for: The benefits and harms of adjuvant chemotherapy for non-small cell lung cancer in patients with major comorbidities: A simulation study
Source: PLoS One. 2022 Nov 15;17(11):e0263911. doi: 10.1371/journal.pone.0263911 (PMC9665372; doi:10.1371/journal.pone.0263911)
Supplement: S4 Table — (DOCX) [file pone.0263911.s004.docx]

**S4 Table.** Percent of scenarios changing conclusions in sensitivity analyses: Varying post-chemotherapy mortality penalty*

| **Stage I** | | **None** | **CAD** | | **CHF** | | **COPD** | | **CAD/CHF** | **CHF/COPD** | **CAD/COPD** |
| --- | --- | --- | --- | --- | --- | --- | --- | --- | --- | --- | --- |
| Male 80-84 years | | 27% | 0% | | 0% | | 27% | | 0% | 0% | 0% |
| Male 75-79 years | | 45% | 27% | | 9% | | 45% | | 0% | 9% | 27% |
| Male 70-74 years | | 36% | 45% | | 18% | | 18% | | 0% | 18% | 45% |
| Male 66-69 years | | 18% | 55% | | 27% | | 9% | | 9% | 27% | 45% |
| Female 80-84 years | | 27% | 9% | | 0% | | 36% | | 0% | 0% | 27% |
| Female 75-79 years | | 36% | 36% | | 18% | | 27% | | 0% | 18% | 45% |
| Female 70-74 years | | 18% | 54% | | 27% | | 9% | | 9% | 27% | 45% |
| Female 66-69 years | | 9% | 45% | | 36% | | 0% | | 9% | 45% | 27% |
|  | |  |  | |  | |  | |  |  |  |
| **Stage IIA** | | **None** | **CAD** | | **CHF** | | **COPD** | | **CAD/CHF** | **CHF/COPD** | **CAD/COPD** |
| Male 80-84 years | | 36% | 9% | | 0% | | 27% | | 0% | 0% | 9% |
| Male 75-79 years | | 36% | 36% | | 18% | | 36% | | 0% | 9% | 36% |
| Male 70-74 years | | 18% | 45% | | 27% | | 9% | | 0% | 27% | 45% |
| Male 66-69 years | | 0% | 36% | | 36% | | 0% | | 9% | 36% | 36% |
| Female 80-84 years | | 36% | 18% | | 0% | | 45% | | 0% | 0% | 18% |
| Female 75-79 years | | 27% | 45% | | 18% | | 18% | | 0% | 27% | 45% |
| Female 70-74 years | | 9% | 36% | | 36% | | 0% | | 9% | 36% | 36% |
| Female 66-69 years | | 0% | 27% | | 45% | | 0% | | 18% | 45% | 18% |
|  | |  |  | |  | |  | |  |  |  |
| **Stage IIB** | | **None** | **CAD** | | **CHF** | | **COPD** | | **CAD/CHF** | **CHF/COPD** | **CAD/COPD** |
| Male 80-84 years | | 36% | 27% | | 9% | | 55% | | 0% | 0% | 18% |
| Male 75-79 years | | 0% | 36% | | 36% | | 0% | | 9% | 27% | 45% |
| Male 70-74 years | | 0% | 9% | | 45% | | 0% | | 27% | 45% | 18% |
| Male 66-69 years | | 0% | 0% | | 27% | | 0% | | 36% | 27% | 0% |
| Female 80-84 years | | 27% | 45% | | 18% | | 27% | | 0% | 18% | 45% |
| Female 75-79 years | | 0% | 18% | | 45% | | 0% | | 27% | 45% | 18% |
| Female 70-74 years | | 0% | 0% | | 27% | | 0% | | 36% | 27% | 0% |
| Female 66-69 years | | 0% | 0% | | 9% | | 0% | | 55% | 9% | 0% |
|  | |  |  | |  | |  | |  |  |  |
| **Stage IIIA** | | **None** | **CAD** | | **CHF** | | **COPD** | | **CAD/CHF** | **CHF/COPD** | **CAD/COPD** |
| Male 80-84 years | | 9% | 55% | | 27% | | 36% | | 0% | 0% | 64% |
| Male 75-79 years | | 0% | 0% | | 36% | | 0% | | 27% | 36% | 0% |
| Male 70-74 years | | 0% | 0% | | 9% | | 0% | | 45% | 36% | 0% |
| Male 66-69 years | | 0% | 0% | | 0% | | 0% | | 27% | 0% | 0% |
| Female 80-84 years | | 0% | 18% | | 45% | | 0% | | 18% | 27% | 36% |
| Female 75-79 years | | 0% | 0% | | 9% | | 0% | | 55% | 27% | 0% |
| Female 70-74 years | | 0% | 0% | | 0% | | 0% | | 27% | 0% | 0% |
| Female 66-69 years | | 0% | 0% | | 0% | | 0% | | 0% | 0% | 0% |
|  | |  | | |  | |  | |  |  |  |
| Base-case conclusion | (no color) | | | Observation | |  | | Adjuvant chemotherapy | | | |

*One-way sensitivity analyses show results of simulations varying parameter for impact of adjuvant chemotherapy on post-chemotherapy mortality. Result is the proportion of simulations where the base-case prediction is maintained when varying the hazard ratio for adjuvant chemotherapy across its 95% confidence range.
